# Immunomodulatory Effects of Juzentaihoto on Fas-Mediated Apoptosis: Insights from Cancer Patients and In Vitro Models

**DOI:** 10.3390/ph18111658

**Published:** 2025-11-01

**Authors:** Quang Trung Ngo, Jorge Luis Espinoza, Hongyang Li, Masafumi Inokuchi, Yosuke Nakanishi, Eriko Morishita, Takamasa Katagiri, Akihiro Kawahara, Tomokazu Yoshizaki, Akiyoshi Takami, Keiko Ogawa-Ochiai

**Affiliations:** 1 Kampo Clinical Center, Hiroshima University Hospital, 1 Chome-2-3 Kasumi, Minami Ward, Hiroshima 734-8551, Hiroshima, Japan; nqtrung@hpmu.edu.vn (Q.T.N.); hoyalee328@yahoo.co.jp (H.L.); abcd@hiroshima-u.ac.jp (A.K.); 2 Faculty of Traditional Medicine, Hai Phong University of Medicine and Pharmacy, Hai Phong 180000, Vietnam; 3 Faculty of Health Sciences, Kanazawa University, Kanazawa 920-0942, Ishikawa, Japan; luis@staff.kanazawa-u.ac.jp; 4 Department of Breast Oncology, Kanazawa Medical University, Kahoku-gun, Kanazawa 920-0293, Ishikawa, Japan; inokuchi@kanazawa-med.ac.jp; 5 Division of Otolaryngology-Head and Neck Surgery, Graduate School of Medical Science, Kanazawa University, Kanazawa 920-8640, Ishikawa, Japan; nakanish@med.kanazawa-u.ac.jp (Y.N.); tomoy@med.kanazawa-u.ac.jp (T.Y.); 6 Department of Clinical Laboratory Science, Graduate School of Medical Science, Institute of Medical, Pharmaceutical and Health Sciences, Kanazawa University, Kanazawa 920-0942, Ishikawa, Japan; eriko86@staff.kanazawa-u.ac.jp (E.M.); takama@staff.kanazawa-u.ac.jp (T.K.); 7 Department of Internal Medicine, Division of Hematology, School of Medicine, Aichi Medical University, Nagakute 480-1195, Aichi, Japan; takami-knz@umin.ac.jp

**Keywords:** juzentaihoto, apoptosis, immune modulation, cancer, Kampo medicine, natural products, traditional medicine

## Abstract

**Background/Objectives:** Juzentaihoto (JTT), a traditional Kampo formula composed of ten medicinal herbs, is widely prescribed in Japan for immune enhancement and general health maintenance. This exploratory, open-label pilot study aimed to evaluate the feasibility and immunomodulatory effects of JTT in cancer patients and to explore its potential mechanisms of action. **Methods:** Ten cancer patients received oral JTT (7.5 g/day) for 14 days, while healthy volunteers served as a reference group. Peripheral natural killer (NK) cell phenotypes and CD95 expression were analyzed by flow cytometry, and serum Fas ligand (FasL) concentrations were measured by ELISA. Complementary in vitro assays using PBS-extracted, autoclaved JTT were conducted to assess Fas/FasL-mediated apoptosis in Jurkat and primary T cells by flow cytometry and Western blotting for cleaved caspase-8 and -3. Additional experiments with staurosporine (intrinsic apoptosis) and TRAIL in OSC-19 carcinoma cells were performed to determine pathway specificity. **Results:** In patients, most NK-cell markers showed no statistically significant within-subject changes, although a trend-level increase in NKp46 and a significant reduction in NK-cell CD95 expression (paired *p* = 0.014) were observed. Between-group differences primarily reflected baseline disparities between cancer patients and healthy controls. In vitro, JTT (50–100 µg/mL) partially attenuated FasL-induced apoptosis and reduced cleaved caspase-3 without affecting cleaved caspase-8, suggesting selective downstream modulation of the extrinsic pathway. **Conclusions:** Within the limitations of a small, non-randomized cohort without placebo, these findings are hypothesis-generating and indicate that JTT selectively modulates Fas-mediated lymphocyte apoptosis without promoting tumor immune evasion. Further randomized trials and mechanistic studies incorporating co-culture or 3D tumor–immune models are warranted to confirm these observations and identify active constituents.

## 1. Introduction

Apoptosis, or programmed cell death, is a vital biological process that maintains tissue homeostasis by eliminating damaged or unnecessary cells during development and aging [1,2]. Dysregulation of apoptosis is a hallmark of many pathological conditions, including cancer [3,4]. Cancer is a leading cause of death worldwide and a major public health concern [5]. Cancer affects individuals across all age groups, with approximately 20,000 adolescents and young adults (aged 15–39 years) diagnosed annually [6], while data from Japan’s national cancer registry showed that 75% of new cases in 2018 occurred in individuals aged 65 years or older, and 44% in those over 75 [7]. In this context, elucidating the pathways that trigger apoptosis will play a pivotal role in designing therapeutic strategies to control cancer development and progression.

Apoptosis can be initiated via two major pathways: the extrinsic pathway, which involves the activation of death receptors such as Fas (CD95) upon ligand binding, and the intrinsic pathway, which is triggered by mitochondrial signals regulated by B-cell lymphoma 2 (BCL-2) family proteins [8,9]. Both pathways converge upon the activation of caspases, particularly caspase-8 and caspase-3, which execute the final phase of apoptosis [8,9,10]. The induction of apoptosis due to Deoxyribonucleic acid (DNA) damage in precancerous lesions can remove potentially harmful cells, thereby blocking tumor growth [11]. Currently, some anti-cancer therapies focus on the extrinsic and intrinsic apoptotic pathways [12,13]. Alternatively, cancer cells can evade apoptosis through aberrantly active survival pathways [14]. Although many types of cancer employ mechanisms to evade apoptosis, certain cancers can actively induce apoptosis in immune cells via the CD95-CD95L pathway as a strategy to escape immune surveillance [15,16]. CD95L (Fas Ligand (FasL)) is also expressed by some cancer cells as a counterattack mechanism to eliminate tumor-targeting immune cells, particularly cytotoxic T cells. When binding to the CD95 (Fas) receptor on the surface of immune cells, CD95L triggers apoptosis through the DISC, similar to what occurs in normal target cells. This mechanism is referred to as “tumor counterattack” [17]. Increased expression of CD95L in the tumor microenvironment may play a direct role in helping the tumor evade immune surveillance, thereby promoting cancer progression.

Harnessing natural compounds to regulate apoptosis offers a promising avenue for improving cancer treatment outcomes and overcoming tumor immune evasion. Juzentaihoto (JTT), also known as Shi-Quan-Da-Bu-Tang, is a traditional Kampo formula consisting of ten medicinal herbs. It is widely used in Japan to treat general fatigue, anemia, and immunodeficiency. In recent years, JTT has shown promising immunomodulatory effects, such as enhancing IL-12 production by dendritic cells, activating natural killer (NK) cells, and promoting IFN-γ secretion [18,19]. In recent years, its application has been extended to cancer therapy, with studies indicating its ability to improve the overall health, physiological condition, and immune function of cancer patients [20,21]. Clinical studies have demonstrated that JTT can improve the quality of life, reduce symptoms such as fatigue, pain, and anxiety in patients with breast cancer [22], and increase peripheral T and B lymphocyte counts in leukemia patients [23]. Furthermore, a retrospective study reported improved prognosis in patients with recurrent cell lung cancer when combined with chemotherapy [24]. Beyond these supportive effects, tumor immune evasion involves apoptosis of immune effector cells via the Fas/FasL (CD95/CD95L) signaling pathway. The FasL, which is expressed on the surface of tumor cells, can interact with Fas (CD95) receptors on immune cells, triggering apoptosis, facilitating immune escape and tumor progression [25,26]. Therefore, the present study aimed to investigate the effects of JTT on FasL-induced apoptosis of immune cells in the tumor microenvironment. Specifically, we evaluated the influence of JTT on lymphocyte viability and immunophenotype in cancer patients, determined the optimal in vitro culture conditions and dosage, and examined its regulatory effects on Jurkat T cell apoptosis through caspase-8 and caspase-3 activation. Clarifying these molecular mechanisms may provide insights into the anti-cancer potential of JTT as an adjunct immunomodulatory therapy. Given the exploratory nature of this research, we designed an open-label pilot study involving a small number of cancer patients without randomization or placebo control, primarily to generate feasibility and mechanistic signals rather than to establish clinical efficacy. Peripheral NK-cell phenotyping was conducted to assess immune modulation in vivo, while in vitro assays using T-cell models (Jurkat and primary lymphocytes) were performed to dissect Fas/FasL-mediated apoptotic mechanisms at the molecular level. The integration of clinical observations and mechanistic experiments in this study was therefore intended to provide hypothesis-generating evidence and a methodological foundation for future randomized controlled and co-culture/3D tumor-microenvironment studies.

## 2. Results

### 2.1. Changes in Immune Cells in Cancer Patients After Using JTT

**Figure 1:**
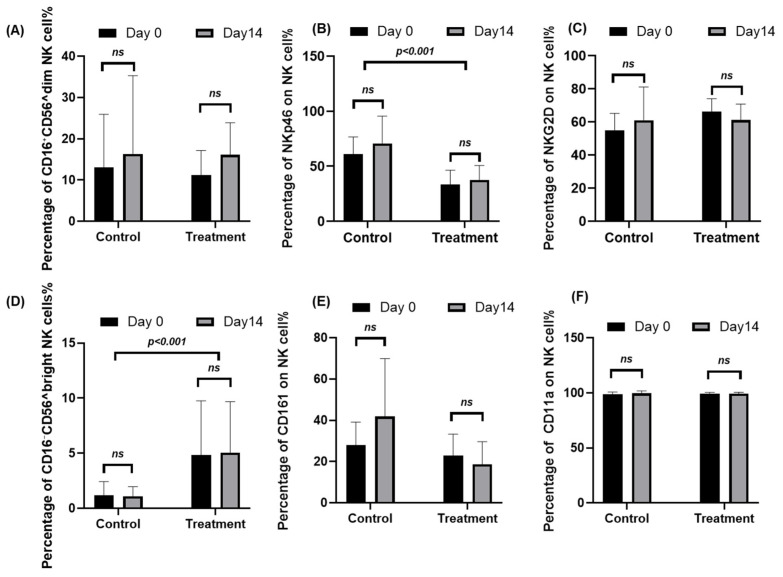
Peripheral blood mononuclear cells (PBMCs) were collected from ten cancer patients at baseline (Day 0) and after 14 days of Juzentaihoto (JTT) treatment (Day 14). For comparison, samples from ten healthy volunteers, who did not receive JTT, were obtained at the same time points (Day 0 and Day 14). Cells were stained with fluorochrome-conjugated antibodies and analyzed by flow cytometry to assess NK-cell subsets and activation/adhesion markers. Panels show the percentage of (**A**) CD56^dim NK cells, (**B**) NKp46^+^ NK cells, (**C**) NKG2D^+^ NK cells, (**D**) CD56^bright NK cells, (**E**) CD161^+^ NK cells, and (**F**) CD11a^+^ NK cells. Data are presented as mean ± SD with 95% confidence intervals (n = 10). Statistical comparisons between Day 0 and Day 14 were performed using two-tailed paired *t*-tests. ns, not significant. Marker expression for NKp46, NKG2D, CD161, and CD11a Panels (**B**,**C**,**E**,**F**) was evaluated in both CD56^dim and CD56^bright NK-cell subsets. Representative gating plots are provided in Supplementary Figure S1, and corresponding numerical data are summarized in Supplementary Table S2.

To evaluate the effects of Juzentaihoto (JTT) on NK-cell phenotypes, peripheral blood samples from ten cancer patients and ten healthy volunteers were analyzed at baseline (Day 0) and after 14 days of JTT treatment. Flow cytometry was used to assess NK-cell subsets (CD56^dim and CD56^bright) and surface expression of activation and adhesion markers. Representative gating plots are provided in Supplementary Figure S1, and quantitative data are summarized in Supplementary Tables S2 and S3. Overall, JTT administration did not induce statistically significant changes in most NK-cell markers (Figure 1). The mean proportion of CD56^dim NK cells increased from 11.15 ± 6.04% to 16.16 ± 7.74% (Δ = +5.01, 95% CI [−2.28, 12.30]; *p* = 0.154), while CD56^bright NK cells changed minimally (4.86 ± 4.90% to 5.06 ± 4.63%; Δ = +0.20; *p* = 0.930). NKp46^+^ NK cells showed a non-significant trend toward higher expression (33.53 ± 12.94% to 37.67 ± 13.02%; Δ = +4.14, 95% CI [−3.87, 12.15]; *p* = 0.272), whereas NKG2D^+^ and CD161^+^ NK cells exhibited mild decreases (Δ = −5.26, *p* = 0.057; Δ = −4.11, *p* = 0.072, respectively). CD11a expression remained stably high (≈99%) before and after treatment (*p* = 0.81). Consistent with these percentage data, MFI values (Supplementary Table S3) showed no significant changes in NKp46, CD161, or CD11a after 14 days. NKG2D MFI decreased modestly (Δ = −641.94, 95% CI [−1226.89, −56.98]; *p* = 0.035), but this trend did not correspond to marked functional shifts in NK-cell activity. Taken together, these findings indicate that short-term JTT administration did not significantly alter NK-cell subset composition or major activation and adhesion markers, although a slight upward trend in NKp46 expression was observed.

### 2.2. Effects of JTT on CD95 Expression in NK Cells

**Figure 2:**
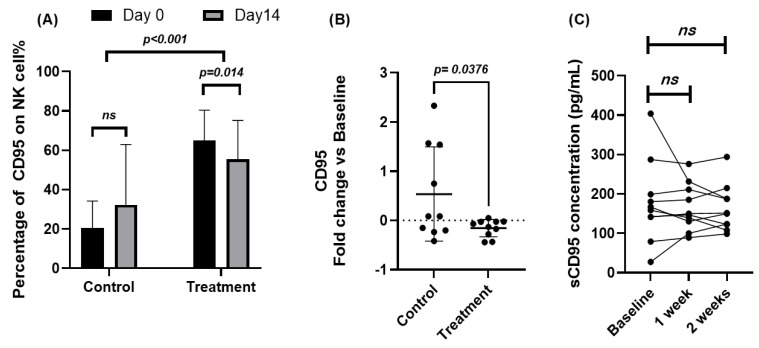
Effects of Juzentaihoto (JTT) on CD95 expression in NK cells and serum Fas ligand (FasL) levels in cancer patients. (**A**) Percentage of CD95^+^ NK cells in ten cancer patients and ten healthy volunteers at baseline (Day 0) and after 14 days of JTT administration, determined by flow cytometry. Statistical comparison between Day 0 and Day 14 was performed using paired *t*-tests. (**B**) Fold change in CD95 expression [(Day 14 − Day 0)/Day 0] in control and patient groups. Between-group comparison was assessed using unpaired *t*-tests. (**C**) Serum Fas ligand (FasL, pg/mL) levels measured by ELISA at baseline, 1 week, and 2 weeks after JTT administration in cancer patients. Each post-treatment time point was compared with baseline using paired *t*-tests. No significant differences were observed in FasL concentrations, with *p* = 0.54 at 1 week and *p* = 0.57 at 2 weeks after JTT administration. Data are expressed as mean ± SD with 95% confidence intervals. ns, not significant; *p* < 0.05, statistically significant.

To evaluate the effects of Juzentaihoto (JTT) on apoptosis-related immune signaling, CD95 expression on NK cells and circulating Fas ligand (FasL) levels were examined by flow cytometry and ELISA, respectively. Quantitative results are summarized in Supplementary Tables S2 and S3. At baseline, cancer patients exhibited markedly higher percentages of CD95^+^ NK cells compared with healthy volunteers (64.91 ± 15.43% vs. 20.53 ± 9.71%). After 14 days of JTT administration, CD95 expression in patients significantly decreased to 55.58 ± 19.64% (Δ = −9.33; 95% CI [−16.31, −2.36]; *p* = 0.014, paired *t*-test), whereas the control group showed a mild but non-significant increase (20.53 → 32.28%; *p* = 0.17). As shown in Figure 2A, this reduction indicates a down-modulation of Fas (CD95) expression following short-term JTT treatment. Between-group comparison of fold-change values confirmed this pattern (Figure 2B): the patient group showed a mean fold-change of −0.156, in contrast to +0.536 in controls (*p* = 0.043, unpaired *t*-test, after ln-transform). Consistent with surface data, the MFI of CD95 also decreased significantly from 3709 ± 1850 to 2982 ± 1194 (Δ = −727; 95% CI [−1313, −141]; *p* = 0.020), further supporting the attenuation of Fas-related signaling (Supplementary Table S3). Serum FasL concentrations, measured at baseline, 1 week, and 2 weeks after JTT administration, showed no significant changes (Figure 2C). Mean ± SD values were 178.5 ± 77.2, 165.9 ± 66.4, and 163.7 ± 59.8 pg/mL, respectively (*p* > 0.5 Vs. Baseline). Nevertheless, the post-treatment variance appeared narrower, suggesting decreased inter-individual variability rather than a consistent directional effect. Collectively, these results indicate that JTT did not alter systemic FasL levels but was associated with a modest, statistically significant reduction of CD95 expression on NK cells in cancer patients, implying possible stabilization of NK-cell survival within an apoptosis-prone immune milieu.

### 2.3. Optimal Conditions for Culturing Jurkat Cell in JTT Medium

Although surface markers such as CD161, NKG2D, and CD11a were also analyzed and showed some positive trends. Their expression levels did not display clear or consistent differences between patients with cancer and healthy controls. In contrast, CD95 expression was significantly altered in patients with cancer treated with JTT compared to that in the control group. Given that CD95 plays a key role in the extrinsic apoptotic pathway of immune cells during anti-tumor responses, a more detailed investigation of JTT’s effect of JTT on this marker is necessary. Accordingly, our research team focused on evaluating the immunomodulatory effects of JTT on CD95 expression and its potential functional consequences. Based on these clinical observations, we conducted in vitro experiments using Jurkat T cells to explore the effects of JTT on immune cell apoptosis. First, to determine the optimal conditions for Jurkat cell culture, we cultured the cells at a density of 1 × 10^6^ cells with various concentrations of JTT: 0 µg/mL, 1 µg/mL, 5 µg/mL, 10 µg/mL, 50 µg/mL, 100 µg/mL, 500 µg/mL, and 1000 µg/mL. The culture was maintained in a 12-well flask for 72 h. After 72 h, images of the cells were captured at 10× and 40× magnifications to observe morphological changes in Jurkat cells.

**Figure 3:**
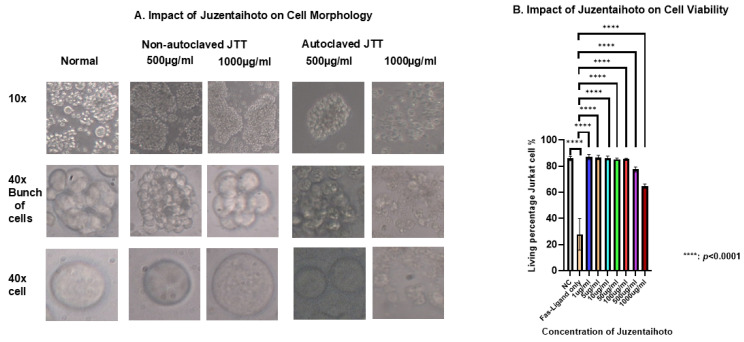

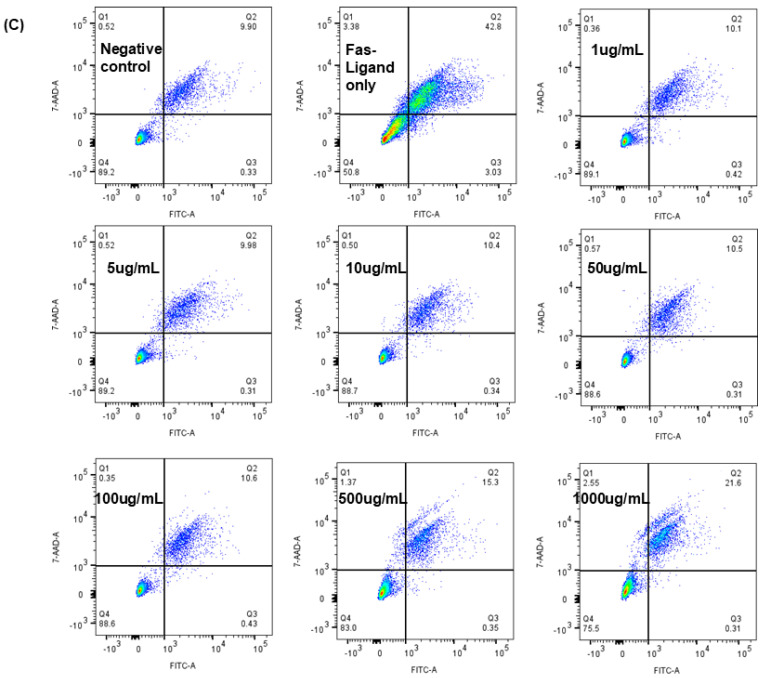
Morphology and viability of Jurkat cells after treatment with various experimental conditions. (**A**) Phase-contrast microscopy images show changes in Jurkat cell morphology, aggregation, and viability under different concentrations of Juzentaihoto (JTT). (**B**) The graph presents the viability rate of Jurkat cells in each treatment group. Data are expressed as mean ± standard deviation from three independent experiments. Statistical analysis was performed using one-way ANOVA followed by appropriate post hoc tests. (**C**) Representative flow cytometry plots showing Fas-Ligand–induced apoptosis in Jurkat cells treated with increasing concentrations of JTT (1–1000 µg/mL). Color density indicates cell population frequency, where blue represents lower and green/yellow represents higher cell density. Cells were stained with annexin V–FITC and 7-AAD to distinguish apoptotic populations. The plots illustrate one representative experiment used for quantitative analysis summarized in (**B**).

In non-autoclaved JTT, the cells tended to aggregate into grape-like clusters, and active cell movement was observed. From 1 µg/mL to 1000 µg/mL, cells maintained a full state and exhibited normal morphology, similar to the negative control. In contrast, with autoclaved JTT, cells cultured between 1 µg/mL and 100 µg/mL also maintained a full state and grape-like clustering. However, at higher concentrations (500 µg/mL and 1000 µg/mL), numerous cell fragments were observed, indicating disruption of cell connectivity. At 1000 µg/mL, Jurkat cells were severely damaged, with numerous cell fragments and dead cells visible. Comparing the two conditions, it was found that non-autoclaved JTT, even at high concentrations, did not exhibit any pharmacological effects, unlike autoclaved JTT. Therefore, subsequent experiments were conducted using autoclaved JTT. To evaluate the effects of autoclaved JTT on Jurkat cells, cells were seeded at a density of 1 × 10^6^ cells/mL into culture plates containing JTT at concentrations of 1 µg/mL, 5 µg/mL, 10 µg/mL, 50 µg/mL, 100 µg/mL, 500 µg/mL, and 1000 µg/mL. Two additional culture plates were used as negative and FasL-only controls, containing only Phosphate-buffered saline (PBS) and culture medium. After 72 h, the cells were harvested and analyzed by flow cytometry to assess their viability. For comparison, the FasL was added to the FasL-only control wells 24 h prior to collection. As shown in Figure 3B, all concentrations of JTT resulted in significantly higher cell viability than the FasL-only control (*p* < 0.0001). When compared to the negative control, which had a viability rate of 85.83%, treatment groups at 1 µg/mL, 5 µg/mL, 10 µg/mL, 50 µg/mL, and 100 µg/mL showed similar or slightly higher viability rates of 86.96%, 86.67%, 86.27%, 85.3%, and 85.43%, respectively. In contrast, the groups treated with 500 µg/mL and 1000 µg/mL showed reduced viability, at 77.5% and 64.73%, respectively.

### 2.4. Effects of Different Concentrations of JTT on Apoptosis in Jurkat Cells

**Figure 4:**
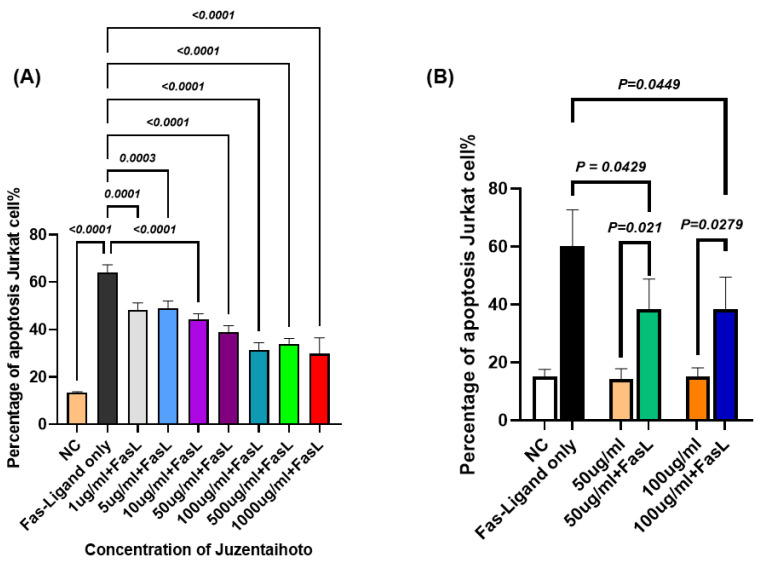
Effect of JTT on FasL-induced apoptosis in Jurkat cells. (**A**) Comparison of Jurkat cell apoptosis between groups treated with different concentrations of JTT, in the presence of FasL. (**B**) Percentage of apoptotic Jurkat cells after FasL stimulation in the presence of various concentrations of JTT (50 µg/mL and 100 µg/mL). NC: negative control (no FasL).

To further investigate whether JTT modulates Fas-mediated apoptosis, we cultured Jurkat cells in the presence of FasL and assessed apoptosis using flow cytometry. Figure 4A shows the apoptosis rate of Jurkat cells cultured in 12-well plates with various concentrations of autoclaved JTT ranging from 1 µg/mL to 1000 µg/mL, as described in the previous experiment. However, 24 h before performing flow cytometry, the FasL was added to all wells, except for the negative control. Flow cytometry was conducted using 7-AAD and ApoTracker Green staining. The apoptosis rate was calculated as the sum of the early and late apoptotic cell percentages based on the flow cytometry results. Figure 4A shows that Jurkat cells treated with different concentrations of JTT exhibited a statistically significant reduction in apoptosis compared to the FasL-only group. Furthermore, the data indicates that the apoptosis rates in the JTT-treated groups were considerably lower, particularly at the concentrations of 50 µg/mL (38.83%), 100 µg/mL (31.4%), 500 µg/mL (34.01%), and 1000 µg/mL (29.83%). However, as shown in the previous figure, the 500 µg/mL and 1000 µg/mL groups also showed a marked decrease in cell viability. Therefore, future experiments will focus on the 50 µg/mL and 100 µg/mL concentrations. In the next experiment, 1 mL of Jurkat cells at a density of 1 × 10^6^ cells/mL was seeded into 6-well flasks. Then, 1 mL of PBS was added to the control group, and JTT at 50 µg/mL and 100 µg/mL was added to the respective treatment groups. The cells were then cultured for 48 h. Twenty-four hours before cell collection, the FasL was added to FasL-only well to serve as a positive control. FasL was also added to one well each of the 50 µg/mL and 100 µg/mL groups, to compare with the same JTT concentrations without FasL. As shown in Figure 4B, the mean percentages of apoptotic Jurkat cells treated with both JTT and FasL at concentrations of 50 µg/mL and 100 µg/mL were 38.53% and 38.58%, respectively. These rates were significantly lower than those in the FasL-only control group, which showed a mean apoptosis rate of 60.16%, with *p* values of 0.0429 and 0.0449, respectively. Although apoptosis decreased after treatment with JTT and the FasL, there was still a clear difference compared to the groups treated with JTT alone. This suggests that JTT partially inhibited the extrinsic apoptotic pathway in Jurkat cells.

### 2.5. Effects of JTT on CD95 Expression in Jurkat Cells at Different Concentrations

**Figure 5:**
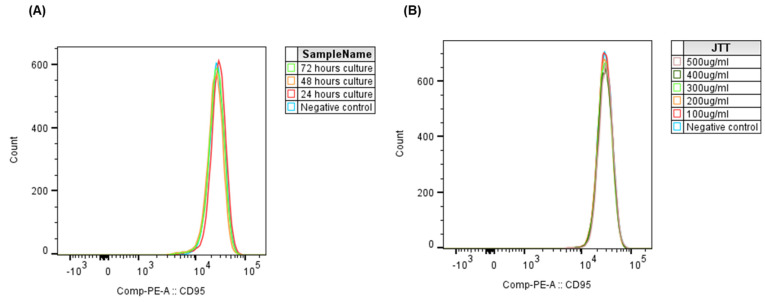
Effect of JTT concentration and culture duration on CD95 expression in Jurkat cells. (**A**) Histogram overlay of CD95 expression in Jurkat cells cultured for 24, 48, and 72 h with 100 µg/mL JTT or PBS as control. (**B**) Histogram overlay showing CD95 expression in Jurkat cells after 48 h of culture with various JTT concentrations (100–500 µg/mL), compared to the PBS control.

To assess whether the duration of culture affects CD95 expression in Jurkat cells, cells were treated with 100 µg/mL JTT and incubated for 24, 48, and 72 h. The control group was cultured in PBS under identical conditions. As shown in Figure 5A, the CD95 expression peak remained stable over time, with no significant differences between time points or compared with the control group (*p* > 0.05). These findings indicate that prolonged culture, with or without JTT, did not alter CD95 surface expression in Jurkat cells.

To further evaluate whether JTT influences CD95 expression in a dose-dependent manner, Jurkat cells were treated with increasing concentrations of JTT (100–500 µg/mL) for 48 h. PBS-treated cells served as a negative control. Flow cytometric analysis revealed no notable shifts in CD95 expression peaks across treatment groups (Figure 5B). This indicates that JTT, even at higher concentrations, did not modulate CD95 expression in Jurkat cells under the present experimental conditions.

### 2.6. Effects of JTT on Apoptosis in T Lymphocytes

**Figure 6:**
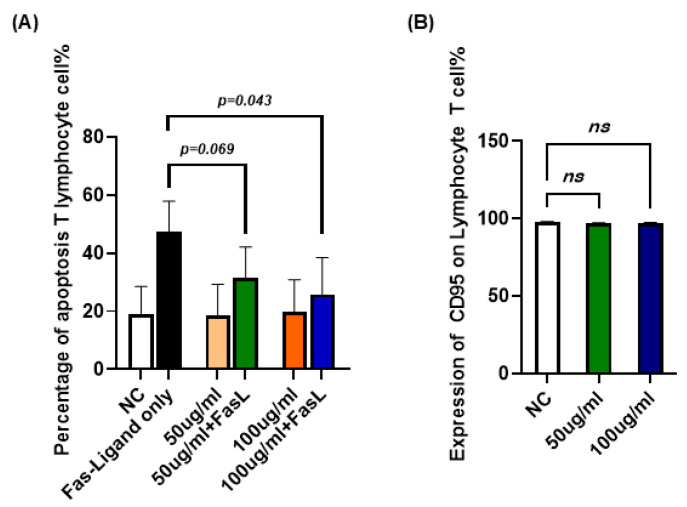
Effect of JTT on FasL-induced apoptosis in human peripheral T lymphocytes. (**A**) Percentage of apoptotic T lymphocytes after treatment with FasL alone (FasL-only), JTT alone (50 µg/mL or 100 µg/mL), or JTT in combination with FasL. NC: negative control (no FasL, no JTT). (**B**) Comparison of the expression of CD95 on T lymphocyte (Negative control, 50 or 100 µg/mL). Values are represented as mean ± SD from independent donors.

To confirm whether the inhibitory effect of JTT on Fas-mediated apoptosis observed in Jurkat cells also occurred in primary human T lymphocytes, we isolated peripheral blood T cells from healthy volunteers. Cells were cultured in RPMI-1640 supplemented with Interleukin-2(IL-2) and subjected to the same apoptosis-inducing conditions used for Jurkat cells. As shown in Figure 6A, treatment with JTT alone (50 or 100 µg/mL) did not significantly alter the baseline level of apoptosis in T lymphocytes compared to the untreated control, indicating no cytotoxicity under these conditions. When the FasL was introduced to induce apoptosis, the addition of JTT resulted in a dose-dependent reduction in the percentage of apoptotic cells. Specifically, the mean apoptotic rate was reduced from the FasL-only group to 31.34% at 50 µg/mL JTT and to 25.89% at 100 µg/mL JTT. However, apoptosis levels in the FasL+JTT group did not return to those observed in cells treated with JTT alone, suggesting that JTT only partially inhibited FasL-induced extrinsic apoptosis. Additionally, as shown in Figure 6B, the expression of CD95 in T lymphocytes remained unchanged compared to that in the negative control group. These findings were consistent with those obtained in Jurkat cells, reinforcing the partial protective effect of JTT against Fas-mediated T-cell apoptosis.

### 2.7. Effects of JTT on Caspase-8 and Caspase-3 Activation in the Extrinsic Apoptosis Pathway

**Figure 7:**
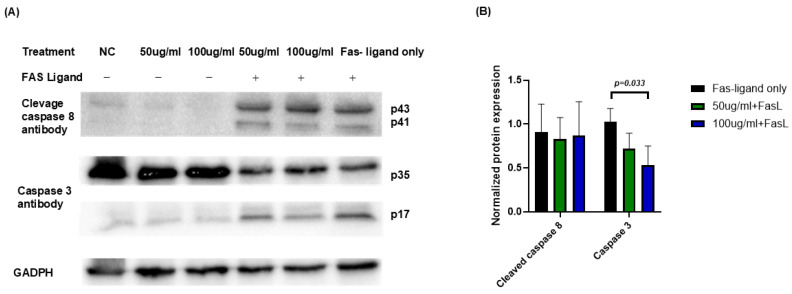
Effect of JTT on activation of caspase-8 and caspase-3 in FasL-stimulated Jurkat cells. (**A**) Western blot analysis of cleaved caspase-8 (p43/p41) and caspase-3 (p17) in Jurkat cells treated with or without FasL in the presence or absence of JTT (50 or 100 µg/mL). GAPDH was used as the loading control. (**B**) Densitometric quantification of cleaved caspase-3 (p17) and cleaved caspase-8 (p43), normalized to GAPDH. Data represents relative intensity compared to the FasL-only control group (FasL only).

To investigate the molecular mechanisms underlying the protective effects of JTT against FasL-induced apoptosis, Jurkat cells were cultured under previously described conditions. On the final day, the cells were harvested, lysed, and the total protein was extracted. Protein concentrations were quantified and equal amounts were subjected to Western blot. Antibodies against cleaved caspase-8 and caspase-3 were used to detect the activation of the extrinsic apoptosis pathway. As shown in Figure 7A, FasL treatment induced cleavage of both caspase-8 (bands at p43/p41) and caspase-3 (band at p17), indicating activation of the apoptotic cascade. In the presence of JTT, especially at 100 µg/mL, the cleaved caspase-3 (p17) band intensity was markedly reduced. At 50 µg/mL, a decreasing trend was observed, though less pronounced. Densitometric analysis (Figure 7B) confirmed that the level of cleaved caspase-3 (p17), normalized to GAPDH, significantly decreased in the 100 µg/mL JTT + FasL group compared to the FasL-only group, while 50 µg/mL showed a modest reduction. These findings suggested that JTT inhibited Fas-mediated apoptosis in Jurkat cells, possibly by attenuating downstream caspase-3 activation.

### 2.8. Effects of JTT on Intrinsic Apoptosis in Jurkat Cells Induced by Staurosporine

**Figure 8:**
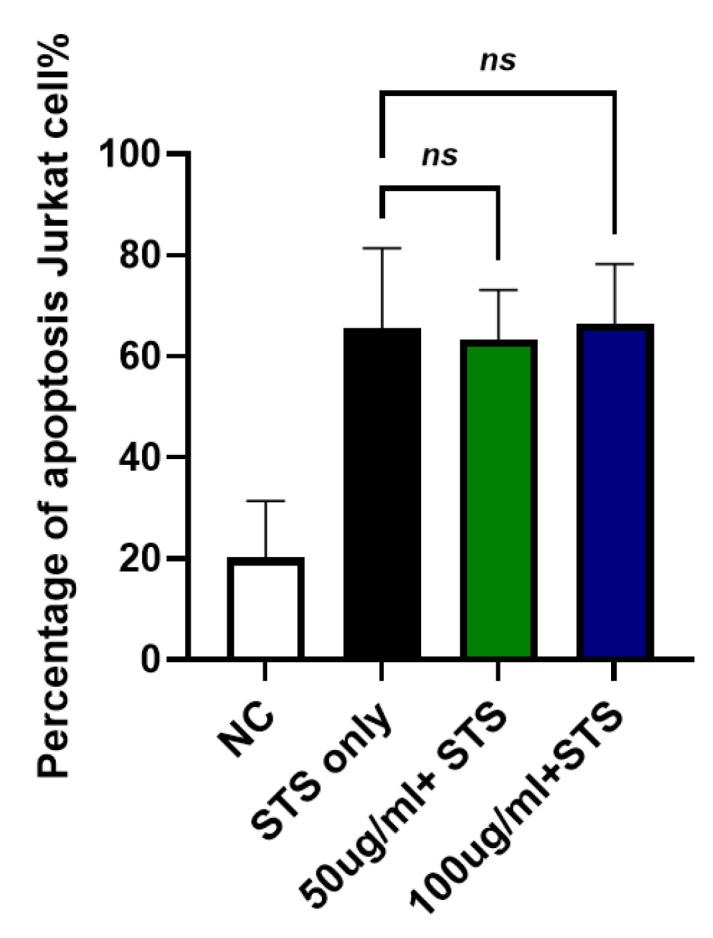
Effect of JTT on intrinsic apoptosis in Jurkat cells. Jurkat cells were cultured under the same conditions as previously described. To induce intrinsic apoptosis, cells were treated with staurosporine instead of FasL. After treatment with JTT (50 µg/mL and 100 µg/mL) or staurosporine only (STS-only), apoptotic cells were quantified by flow cytometry. Data represents mean apoptosis percentage from three independent experiments. No significant difference was observed between JTT-treated groups and STS-only, indicating that JTT does not inhibit intrinsic apoptosis.

To investigate whether JTT affects intrinsic apoptosis, Jurkat cells were treated with staurosporine, a known inducer of the intrinsic apoptotic pathway, instead of FasL. As shown in Figure 8, staurosporine treatment (STS-only) markedly increased apoptosis (mean: 65.50%). However, the addition of JTT at both 50 µg/mL (63.29%) and 100 µg/mL (66.45%) did not significantly alter apoptosis levels compared to the STS-only group. These results suggested that JTT did not inhibit intrinsic apoptosis in Jurkat cells.

### 2.9. Effects of JTT on TRAIL-Induced Apoptosis in Cancer Cells

**Figure 9:**
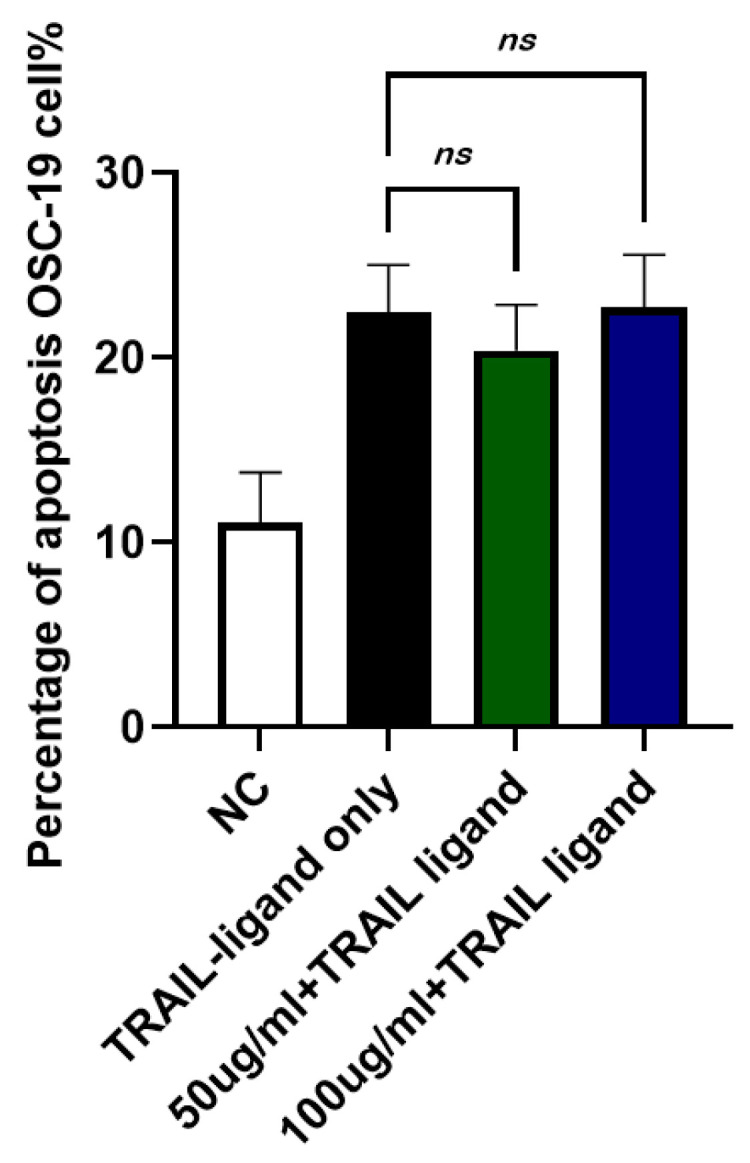
Effect of JTT on TRAIL-induced apoptosis in OSC-19 cancer cells. OSC-19 cells were cultured in DMEM and treated with TRAIL ligand to induce extrinsic apoptosis. JTT was administered at 50 µg/mL or 100 µg/mL and cells were incubated for 48 h. Apoptosis was evaluated by flow cytometry after 24 h of TRAIL exposure. Data represents the mean apoptotic cell percentage from three independent experiments. No significant reduction in apoptosis was observed in the JTT-treated groups, indicating that JTT does not inhibit apoptosis in this cancer cell line.

To examine whether JTT could suppress apoptosis in cancer cells, the human oral squamous carcinoma cell line, OSC-19, was used. Cells were cultured in DMEM and treated with TRAIL ligand, which mimics FasL in inducing extrinsic apoptosis in certain cancer cells, for 24 h. JTT was added at concentrations of 50 µg/mL and 100 µg/mL. After 48 h, the number of apoptotic cells was assessed using flow cytometry. As shown in Figure 9, the TRAIL ligand alone (TRAIL-ligand only) induced apoptosis by 65.50%. The addition of JTT at 50 µg/mL (63.29%) or 100 µg/mL (66.45%) did not reduce apoptosis levels, suggesting that JTT does not inhibit TRAIL-induced apoptosis in OSC-19 cancer cells.

## 3. Discussion

In this study, we explored that the traditional multi-herbal formulation JTT exerts potent immunomodulatory effects in patients with cancer. Consistent with the immunoregulatory properties of Kampo medicine [27,28], JTT administration was associated with a modest trend toward enhanced natural killer (NK) cell activity. Although the changes were not statistically significant, we observed a tendency toward increased frequencies of NKp46+ NK cells and CD56^dim and CD56^bright subsets following JTT treatment. In our previous study in non-cancer patients with fatigue, significant increases in CD56^dim and NKp46 expression were detected after JTT administration [29]. The attenuated or variable response in patients with cancer may be due to immune suppression driven by inhibitory cytokines and TME signals from the tumor microenvironment [30]. Additionally, significant baseline differences between patients with cancer and healthy controls were consistent with prior studies on CD56bright NK cell percentages [31,32] and NKp46 expression [33,34]. NKp46, a key activating receptor in the immunoglobulin superfamily, plays a crucial role in tumor recognition and elimination via antibody-independent mechanisms [35]. Recently, it has been proposed as a promising biomarker for cancer immunotherapy because of its regulatory role in NK cell cytotoxicity and cytokine production [36]. Multiple studies are currently developing NKp46-targeted therapies to improve anti-tumor efficacy [37,38]. Thus, the modest upregulation of NKp46 may represent a positive immunological signal, thereby supporting its potential application in cancer treatment. Importantly, JTT has been safely administered to patients with cancer without serious adverse effects, and this immune stimulation has been linked to improved clinical outcomes, such as better chemotherapy tolerance and reduced treatment-related side effects, without compromising anti-tumor efficacy [24,25,26,27,28,29,30,31,32,33,34,35,36,37,38,39]. Therefore, the subtle enhancement of NK-cell function may contribute to the supportive or adjunctive immunological effects of JTT reported in earlier research.

While the expression levels of receptors such as CD11a and CD161 remained largely unchanged, a notable finding was the sustained reduction in NKG2D and CD95 expression in NK cells after 14 days of JTT administration. Although the decrease in NKG2D was observed only in its MFI without a significant change in the percentage of NKG2D^+^ NK cells, this pattern suggests a per-cell down-modulation rather than a loss of the NKG2D-positive subset. Given that NKG2D can be chronically upregulated under immune stress or aging conditions (e.g., due to persistent ligand engagement or DNA damage responses) [40], its normalization following JTT may reflect a balanced immunomodulatory effect, preventing overactivation and functional exhaustion of NK cells within the tumor-related immune environment. This immunoregulatory action aligns with the concomitant decrease in CD95 expression, suggesting that JTT attenuates activation-induced cell death, thereby reducing NK cell apoptosis—a phenomenon also noted in our previous study [29]. Downregulation of CD95 enhances the stability and persistence of NK cell populations within the immunosuppressive tumor microenvironment [41], which is known to induce Fas/FasL-mediated apoptosis in immune cells [42]. Therefore, although JTT may not uniformly affect all NK cell subsets, it appears to exert a distinct regulatory effect that stabilizes NK cell function through the modulation of both NKG2D and CD95 expression.

The immunological effects of JTT may be partly attributed to the bioactive properties of the individual herbal components. Astragalus membranaceus, a key ingredient, has been shown to enhance macrophage and dendritic cell function and promote Th1 cytokine production, thereby supporting cell-mediated immune responses and inhibiting tumor progression [43]. Ginseng contains ginsenosides that stimulate NK cell cytotoxicity and modulate apoptosis-related pathways such as PI3K/Akt and MAPK signaling, offering both immunostimulatory and anti-apoptotic benefits [44,45]. Furthermore, Angelica sinensis improves hematopoiesis, enhances IL-2 production, and protects lymphocytes from chemotherapy-induced apoptosis, potentially maintaining immune homeostasis during cancer treatment [46]. These cumulative effects of multiple herbs may explain the observed modulation of NK cell phenotype and apoptosis-related markers in this study. Because JTT is a multicomponent formulation, identifying its principal active constituents remains challenging. The observed effects are likely derived from synergistic interactions among multiple herbal ingredients rather than from a single compound. Conducting bioactivity-guided fractionation or testing individual herbs—such as *Panax ginseng* and *Astragalus membranaceus*—may help elucidate the key bioactive molecules, which represents a central objective of our future research.

We also established optimized preparation conditions for in vitro experiments. Specifically, JTT must be autoclaved in PBS and used at concentrations below 100 µg/mL. This method is simple, reproducible, and preserves the biological activity of the JTT components without inducing cytotoxicity at working doses. These features are consistent with those of previous reports emphasizing the importance of autoclaving in herbal extract research [47,48,49]. Prior studies have confirmed that JTT at concentrations under 100 µg/mL is non-toxic and retains immunomodulatory effects in immune cell models [50,51]. Additionally, using PBS as a solvent helps maintain preparation stability, while avoiding potential bioactivity interference from organic solvents.

Furthermore, our results showed that JTT partially inhibited Fas-induced apoptosis via the extrinsic pathway in both human donor-derived T lymphocytes and Jurkat cells, a well-established T cell model for studying immune regulation and TCR signaling [52]. Fas (CD95) is a death receptor belonging to the TNFR family. Upon FasL binding, it recruits FADD to form the DISC, triggering caspase-8 activation [53], which subsequently activates caspase-3, leading to hallmark apoptotic features, such as DNA fragmentation and nuclear condensation [54,55]. Interestingly, our Western blot data revealed that, while caspase-8 was normally activated, cleaved caspase-3 levels were significantly reduced following JTT treatment. This suggests that JTT may intervene downstream of caspase-8, possibly by modulating intermediate regulatory molecules.

Possible mechanisms include interference with members of the Inhibitor of Apoptosis Protein (IAP) family, especially XIAP, which directly inhibits caspase-3 and caspase-7 without affecting caspase-8 [56,57]. Other molecules, such as cIAP1 and cIAP2, may exert indirect regulation via signaling complexes or the NF-κB pathway [58]. Although FLIP primarily inhibits caspase-8 in DISC, it also regulates downstream apoptotic propagation [59]. Intracellular stress and activation of protective pathways such as NF-κB or MAPK may also reduce downstream caspase activation, including caspase-3 [60]. Therefore, JTT may indirectly suppress the extrinsic apoptotic cascade beyond caspase-8 through a combination of its herbal constituents, reflecting a finely tuned immunoregulatory profile. This partial inhibition may selectively modulate the extrinsic pathway, while preserving apoptosis in other contexts, possibly protecting T lymphocytes from excessive death during immune stress, a phenomenon described in recent studies on Kampo formulations [29,30,31,32,33,34,35,36,37,38,39,40,41,42,43,44,45,46,47,48,49,50,51,52,53,54,55,56,57,58,59,60,61].

To further investigate pathway specificity, we used intrinsic pathway inducers, such as staurosporine, which triggers apoptosis via mitochondrial cytochrome c release and caspase-9 activation [62]. JTT did not alter the apoptosis rates induced by these agents, indicating specificity for the extrinsic pathway and lack of interference with the intrinsic apoptotic machinery. Intrinsic apoptosis proceeds independently of DISC and includes the release of Smac/DIABLO, which neutralizes IAPs such as XIAP, cIAP1, and cIAP2 [63], thereby relieving the inhibition of caspase-9 and caspase-3 [64]. The preservation of this pathway may provide a safety margin by maintaining its ability to eliminate DNA-damaged or transformed cells [65,66].

We further examined whether JTT affects carcinoma cells using the oral squamous cell carcinoma line OSC-19 stimulated with TRAIL, which binds to DR4/DR5 and activates extrinsic apoptosis via DISC and caspase-8 [67,68]. JTT did not reduce TRAIL-induced apoptosis in OSC-19 cells, suggesting that it had no protective effect on cancer cells. This is an important finding because the suppression of apoptosis in tumor cells is undesirable. This observation aligns with those of previous studies, indicating that herbal immunomodulators can selectively regulate immune cell apoptosis without interfering with tumor cell susceptibility. For instance, the traditional Chinese formula Yang Wei Kang Liu, containing ginseng, atractylodes, and licorice, has been shown to modulate Fas/FasL expression in T lymphocytes and reduce FasL mRNA levels in gastric carcinoma cells [69,70]. These data underscore the potential safety of JTT as a selective immunomodulatory agent that does not promote immune evasion in tumor settings.

When evaluating the immunomodulatory effects of JTT in cancer patients, it is crucial to consider background factors that may confound systemic immune status. Analysis of 10 patient cases revealed several frequently observed potential confounders, including age ≥ 65, history of chemotherapy and/or radiotherapy, use of hormonal agents (such as tamoxifen, letrozole, TS-1), chronic comorbidities (e.g., diabetes, COPD, heart failure), low BMI (<21), and nutritional support via feeding tubes (Percutaneous Endoscopic Gastrostomy (PEG) or nasogastric). Among these, chemoradiotherapy can suppress immune function by inhibiting hematopoiesis and damaging the mucosal barriers [71,72], which are associated with chronic inflammation and hypoalbuminemia, both of which impair cellular immunity [73]. Hormonal therapies may modulate lymphocyte activity via the cytokine pathways [74]. Chronic conditions such as COPD and diabetes can also contribute to long-term immune dysregulation [75,76]. Notably, enteral feeding may disrupt the gut–immune axis [77], which is one of the potential sites of JTT action. The simultaneous presence of multiple confounding factors complicates the accurate assessment of JTT’s immunomodulatory efficacy of JTT, highlighting the need for controlled clinical studies with appropriate stratification to minimize bias and ensure reliable conclusions. In this cohort, the degree of clinical heterogeneity was considerable, with an average of nearly six confounders per patient (Supplementary Table S1). Some patients exhibited up to nine pre-treatment burdens, including repeated chemoradiation exposure, hormonal therapy, metabolic or cardiovascular comorbidities, and nutritional compromise. Such overlapping immunosuppressive conditions likely attenuate the measurable immune response to JTT, masking its potential biological effects. Therefore, the observed immune variability may reflect intrinsic host factors rather than direct pharmacologic modulation. Recognizing and quantifying these confounding elements is essential for interpreting Kampo-related immunological outcomes and for designing future stratified randomized trials that can more accurately delineate JTT’s mechanistic contribution.

Despite providing novel insights, this study has several limitations. The small, open-label, non-placebo-controlled cohort limits generalizability, and the inclusion of both cancer patients and healthy volunteers introduced inherent biological heterogeneity that reflects genuine immune differences rather than treatment effects. In addition, a preparation mismatch existed between the clinical and in vitro components—patients received the standard non-autoclaved formulation, whereas autoclaved, PBS-extracted JTT was used for cell assays—which may have altered heat-labile constituents and affected pharmacological comparability. The in vitro models also lacked the complexity of the tumor microenvironment. Future studies should address these issues through larger randomized controlled trials, head-to-head comparisons of autoclaved and non-autoclaved extracts, bioactivity-guided fractionation to identify active components, and the use of co-culture or 3D tumor–immune models to clarify the mechanistic and clinical relevance of JTT.

**Figure 10:**
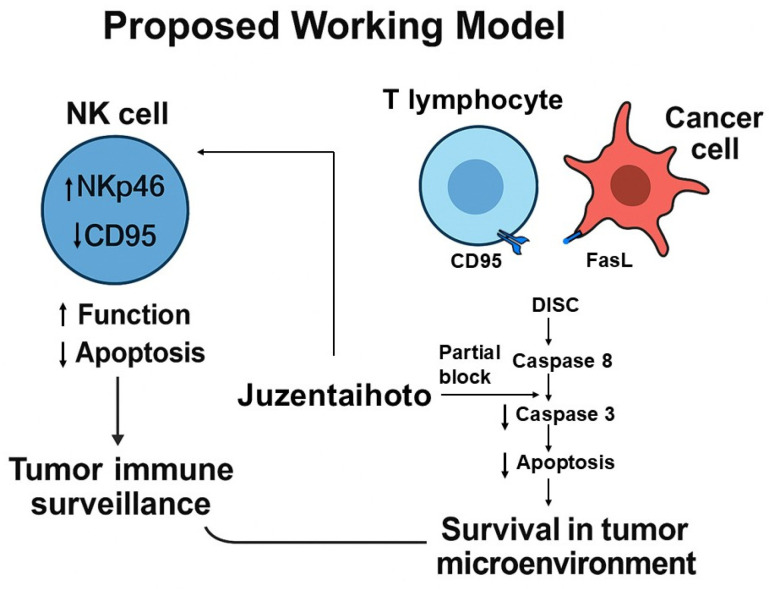
Proposed working model of Juzentaihoto (JTT)–mediated immunomodulation in the tumor microenvironment.

In summary, JTT activates NK cells and selectively modulates Fas-mediated extrinsic apoptosis in lymphocytes while preserving both intrinsic and TRAIL-induced apoptosis in carcinoma cells. Selective immunoregulation may provide clinical benefits by preventing excessive T cell apoptosis, which contributes to T cell exhaustion and immune suppression in chronic diseases and cancers [78,79]. By attenuating this pathway, JTT may enhance T cell persistence and immune responsiveness in disease contexts where lymphocyte survival is critical [80,81]. These findings are conceptually summarized in the proposed mechanistic model (Figure 10)

## 4. Materials and Methods

### 4.1. Clinical Study

#### 4.1.1. Participants

This was a single-arm, non-placebo pilot; Twenty participants were recruited to participate in this study. The control group consisted of 10 individuals (3 males and 7 females) with a mean age of 33.9 years. This group was established to monitor whether the NK cells maintained their functional capacity over time. The treatment group, which received the Kampo medicine JTT, also comprised 10 patients (four males and six females), with detailed patient information presented in Table 1. To assess the potential confounding factors that may influence the therapeutic efficacy of juzentaihoto (JTT) for cancer treatment, we compiled detailed clinical data from 10 patients and analyzed the presence of nine commonly encountered clinical variables. These factors included age > 65 years, prior history of cancer, previous chemotherapy, radiotherapy, history of major or multiple surgeries, presence of comorbidities, low body mass index (BMI < 21), use of immunosuppressive or hormonal agents, and the need for nutritional support via percutaneous endoscopic gastrostomy (PEG) or nasogastric tube. As summarized in Supplementary Table S1, each factor was recorded as either present (“True”) or absent (“False”). Detailed prior therapies, concomitant medications, comorbidities, and baseline immune parameters were systematically abstracted and are provided in Supplementary Table S1. These variables were considered in sensitivity descriptions acknowledging potential confounding. The total number of confounding factors was calculated for each patient, revealing that most individuals exhibited multiple coexisting risk factors, ranging from four to nine per case. Notably, patients 7 and 10 showed the highest number of confounding factors (7 and 9, respectively), including advanced age, prior malignancies, multimodal treatments (combining radiotherapy, chemotherapy, and hormone therapy), comorbidities, and enteral nutritional support. These findings indicate a substantial degree of clinical heterogeneity within the study population, with many patients carrying a significant pretreatment burden before the initiation of JTT.

The study protocol was approved by the Ethics Committee of Kanazawa University and registered under UMIN ID: UMIN000028094, available online at: https://center6.umin.ac.jp/cgi-open-bin/ctr/ctr_view.cgi?recptno=R000032164 (accessed on 31 September 2025).

##### JTT Treatment 

Juzentaihoto (JTT; TJ-48, Tsumura & Co., Tokyo, Japan) was administered orally at a dose of 7.5 g/day for 14 days. This Kampo formula, approved by the Japanese Ministry of Health, Labour and Welfare in 1986, is regulated by the Society of Japanese Oriental Medicine (see http://mpdb.nibiohn.go.jp/stork) (accessed on 31 September 2025). JTT extract granules are manufactured by Tsumura under GMP (Good Manufacturing Practice) standards and, according to the official package insert, are not autoclaved in the clinical formulation. Healthy volunteers did not receive JTT and provided time-matched reference blood samples.”

##### Flow Cytometry

Peripheral blood mononuclear cells (PBMCs) were isolated from whole blood collected on days 0, 7, and 14 using density-gradient centrifugation. Erythrocytes were lysed with an ammonium chloride-based buffer (8.26 g/L NH_4_Cl, 1.0 g/L KHCO_3_, and 0.037 g/L EDTA–4Na) and washed twice with saline supplemented with 10% fetal bovine serum (FBS). For surface immunophenotyping, cells were stained with fluorochrome-conjugated monoclonal antibodies targeting activation markers CD335 (NKp46), CD161, and CD314 (NKG2D); adhesion marker CD11a; and apoptosis marker CD95 (Fas). All antibodies were obtained from BioLegend (San Diego, CA, USA). Staining was performed according to the manufacturer’s instructions in the dark at 4 °C for 30 min, followed by washing with phosphate-buffered saline (PBS) containing 1% FBS. Data acquisition was conducted on a BD FACSCanto II flow cytometer (BD Biosciences, San Jose, CA, USA), and fluorescence compensation was performed using single-stained controls. At least 10,000 CD3^−^CD56^+^ events were collected per sample. Data were analyzed with FlowJo software (version 10; Ashland, OR, USA). Natural killer (NK) cells were identified as CD3^−^CD56^+^ lymphocytes after sequential gating of singlets and lymphocyte populations based on forward and side scatter (FSC/SSC) parameters. NK cells were further divided into CD56^bright CD16^−^ (cytokine-producing) and CD56^dim CD16^+^ (cytotoxic) subsets. The gating strategy followed the sequential order: singlets → lymphocyte gate → CD3^−^CD56^+^ → CD56^bright/CD16^−^ and CD56^dim/CD16^+^, with representative plots provided in Supplementary Figure S1. The expression of activation (CD335, CD161, CD314), adhesion (CD11a), and apoptosis (CD95) markers was evaluated in each subset. Both percentage of positive cells and mean fluorescence intensity (MFI) values were calculated to assess NK-cell activation, adhesion, and apoptotic susceptibility. Percentage and MFI data for each marker and time point (Day 0 and Day 14) are summarized in Supplementary Table S2 (Percentage) and Supplementary Table S3 (MFI). Data acquisition was performed immediately after staining using FACSDiva software v9.0 (BD Biosciences), and results were expressed as mean ± SD.

##### Quantification of FasL by ELISA on Cancer Patients

The concentration of the FasL in patient serum samples was measured using the Human Fas Ligand/TNFSF6 Quantikine ELISA Kit (Cat. No. DFL00B; R&D Systems, Minneapolis, MN, USA) according to the manufacturer’s instructions. Each 50 μL serum sample was added to wells pre-coated with a monoclonal antibody specific to human FasL. After 2 h of incubation at room temperature, the wells were washed to remove any unbound substances. A conjugated detection antibody was added, and the cells were incubated for another 2 h at room temperature. After washing, a substrate solution was added, and color development proceeded for 30 min. The reaction was stopped with a stop solution, and the optical density (OD) was measured at 450 nm with wavelength correction at 540 nm using a microplate reader. The FasL concentrations were determined by comparison with a standard curve generated using recombinant human FasL standards ranging from 15.6 to 1000 pg/mL. The minimum detectable dose (sensitivity) for this assay was 5.1 pg/mL. All samples were analyzed in a paired fashion to compare the serum FasL levels before and after treatment.

### 4.2. In Vitro Study

#### 4.2.1. Cells

In this study design, peripheral NK-cell profiling was conducted in vivo to assess immune modulation in a clinically relevant context using limited blood samples, whereas in-vitro experiments focused on the Fas/FasL signaling pathway in T cells (Jurkat and primary human T lymphocytes). This strategy was chosen because NK-cell function can be evaluated directly from patient blood, while detailed mechanistic interrogation of Fas-mediated apoptosis—including caspase-8 and caspase-3 activation—requires controlled cellular systems that are experimentally tractable. Therefore, the in vivo and in vitro components were designed to address complementary aspects of immune regulation: population-level modulation of NK-cell phenotypes in patients, and pathway-level mechanistic validation of apoptosis control in T cells. The Jurkat cells were kindly provided by Professor Espinoza at Kanazawa University. Primary T lymphocytes were isolated directly from the peripheral blood donated by healthy volunteers. OSC-19 cells were purchased from the JCRB Cell Bank (JCRB0197; Lot No. 05112018, Osaka, Japan).

#### 4.2.2. Cell Culture

Jurkat cells were cultured in RPMI-1640 medium supplemented with l-glutamine (Fujifilm Wako Pure Chemical Corporation, Osaka, Japan, [W01W0118-0215]), 10% fetal bovine serum (FBS; Thermo Fisher Scientific, Waltham, MA, USA), and 1% antibiotics (Nacalai Tesque, Kyoto, Japan). The cells were maintained in 25 mL flasks and monitored weekly. All cultures were incubated at 37 °C in a humidified atmosphere containing 5% CO_2_.

Primary T-lymphocytes were obtained by isolating peripheral blood mononuclear cells (PBMCs) via density-gradient centrifugation. After removing the plasma layer and washing with PBS, cells were incubated with Dynabeads Human T-Activator CD3/CD28 (Thermo Fisher Scientific, Waltham, MA, USA) for activation and expansion. The cells were then cultured in RPMI-1640 medium supplemented with 10% FBS, 1% antibiotics, and 20 ng/mL recombinant human interleukin-2 (IL-2; PEPROTECH, Cranbury, NJ, USA). On day 3, 2 mL fresh culture medium was added, resulting in a total volume of 4 mL. The cells were used for experiments on day 7. All cultures were maintained at 37 °C with 5% CO_2_.

#### 4.2.3. JTT Preparation

The JTT extract powder (Lot No. 2220048010) was provided by Tsumura & Co. (Tokyo, Japan). For in vitro experiments, the powder was suspended in phosphate-buffered saline (PBS), autoclaved at 121 °C for 20 min to ensure sterility and standardization, then filtered through a 0.45 μm Omnipore™ PTFE membrane (Merck Millipore, Merck Millipore, Burlington, MA, USA, Cat. No. JHWP04700, Lot No. R1DB88618) and diluted to the desired concentrations. Pilot observations revealed morphological differences compared with non-autoclaved powder; therefore, all mechanistic assays employed a single autoclaved PBS-extract to minimize batch variability. We acknowledge that patients receive non-autoclaved JTT in clinical settings; hence, direct translational equivalence is limited and should be addressed in future studies comparing matched non-autoclaved and heat-treated preparations.

### 4.3. Experimental Procedures

#### 4.3.1. Morphological Observation

Jurkat cells were cultured in 12-well plates with JTT for three days. Morphological evaluation was performed under a light microscope at 10×, 20×, and 40× magnifications. Comparisons were made between Autoclaved and non-autoclaved JTT treatments were compared.

#### 4.3.2. Flow Cytometry

After 3 days of culture with JTT, Jurkat cells and T lymphocytes were harvested, centrifuged, and washed with PBS. The cells were stained with the following antibodies: CD95, CD3, 7-AAD, and Apotracker Green (all purchased from BioLegend) and analyzed by flow cytometry. Flow cytometry results were analyzed using quadrant gating, where Q4 represented viable cells (Apotracker Green-negative, 7-AAD-negative), Q3 represented early apoptotic cells (Apotracker Green-positive, 7-AAD-negative), Q2 represented late apoptotic or necrotic cells (Apotracker Green-positive, 7-AAD-positive), and Q1 represented necrotic cells (Apotracker Green-negative, 7-AAD-positive). The total number of apoptotic cells was calculated as the sum of the Q2 and Q3 cells.

#### 4.3.3. Induction of Extrinsic Apoptosis

To induce extrinsic apoptosis, Jurkat and T lymphocyte cells were seeded in 6-well plates and treated with JTT on day 1. On day 2, 80 µg/mL Fas ligand (recombinant human TNFSF6, BioLegend) was added. Apoptosis was assessed after 24 h.

#### 4.3.4. Induction of Intrinsic Apoptosis

To evaluate the effect of JTT on intrinsic apoptosis, Jurkat cells were seeded in 6-well plates and treated with JTT. On the following day, 1 uM staurosporine (Sigma-Aldrich, St. Louis, MO, USA; Lot No. 0000090770) was added to induce apoptosis. After 24 h of incubation, cells were harvested for analysis.

#### 4.3.5. Induction of Extrinsic Apoptosis in Cancer Cells

OSC-19 cells were cultured in 6-well plates for 48 h, and JTT was added at the beginning of the culture. After 24 h, 100 ng/mL recombinant human TRAIL ligand (TNFSF10, carrier-free; BioLegend) was added to induce apoptosis. After 48 h, the cells were harvested for analysis using trypsin-EDTA (0.25%) with phenol red (Thermo Fisher Scientific) to, which enzymatically dissociates adherent cells from the culture surface.

#### 4.3.6. Western Blot Analysis

Jurkat cells were harvested and washed twice with ice-cold phosphate-buffered saline (PBS) by centrifugation at low speed (4 °C). The resulting cell pellet was resuspended in ice-cold RIPA buffer supplemented with protease inhibitors (RIPA: PMSF = 100:1) at a ratio of 1 mL buffer per 1 × 10^7^ cells. The suspension was incubated on ice for 30 min. To disrupt genomic DNA and cellular components, lysates were briefly sonicated on ice for 15–30 s. The lysates were clarified by centrifugation at 12,000 rpm for 10 min at 4 °C. The resulting supernatant containing total protein was collected and stored on ice for immediate use, or at −20 °C or −80 °C for later analysis. The protein concentration was determined using a DC Protein Assay Kit (Bio-Rad Laboratories, Hercules, CA, USA) according to the manufacturer’s instructions.

Equal amounts of protein were separated on 12% SDS-PAGE gels and transferred to PVDF membranes using a wet transfer system at 100 V for 1 h at 4 °C. Membranes were blocked and incubated overnight at 4 °C with primary antibodies against cleaved caspase-8, cleaved caspase-3, and GAPDH (all from Cell Signaling Technology, Danvers, MA, USA). After washing, the membranes were incubated with horseradish peroxidase-conjugated secondary antibodies (anti-rabbit IgG, horseradish peroxidase-linked antibody and anti-biotin, horseradish peroxidase-linked antibody; Cell Signaling Technology) for 1 h at room temperature. Protein bands were visualized using an enhanced chemiluminescence (ECL) detection kit (Clarity™ Western ECL Substrate, Bio-Rad Laboratories, Inc., Hercules, CA, USA), and signals were captured using the ChemiDoc™ MP Imaging System (Bio-Rad Laboratories, Hercules, CA, USA).

#### 4.3.7. Image Analysis

Western blot images were processed using the ImageJ software (version 1.53t, 2022).

### 4.4. Statistical Analysis

Clinical study: For flow cytometry data, the expression levels of immune markers (e.g., CD95, CD161, NKp46) before and after treatment were compared using two-tailed paired *t*-tests. For ELISA results (e.g., serum FasL levels), post-treatment values were compared with baseline using paired *t*-tests. Fold-changes between cancer patients and healthy controls were analyzed after natural log transformation [ln(Post/Pre)] using unpaired *t*-tests. Data normality was assessed with the Shapiro–Wilk test; when normality assumptions were violated, non-parametric alternatives (Wilcoxon signed-rank or Mann–Whitney U tests) were applied. Effect sizes (Hedges’ *g* for parametric and rank-biserial correlation for non-parametric tests) and 95% confidence intervals (CIs) were also reported. For multiple immune markers, *p*-values were adjusted using the Benjamini–Hochberg false discovery rate (FDR) procedure.

In vitro study: For in vitro experiments (Jurkat cells and primary lymphocytes), comparisons between the treatment and control groups were performed using one-way ANOVA, followed by Dunnett’s multiple comparison test. For paired comparisons under identical treatment conditions (e.g., with/without FasL stimulation), paired *t*-tests were used. If data were not normally distributed, Kruskal–Wallis and Wilcoxon signed-rank tests were applied as non-parametric alternatives. All results are presented as mean ± standard deviation (SD) unless otherwise indicated. A *p*-value < 0.05 (after FDR correction when applicable) was considered statistically significant. All statistical analyses were performed using GraphPad Prism version 9.5.1 (GraphPad Software, San Diego, CA, USA).

## 5. Conclusions

Within the constraints of an exploratory design, JTT was associated with a significant reduction in NK-cell CD95 in vivo and a selective attenuation of Fas-mediated apoptosis downstream of caspase-8 in T cells in vitro, without affecting intrinsic apoptosis or TRAIL-induced cancer-cell apoptosis.

## Figures and Tables

**Table 1:** JTT treatment patient characteristics.

Characteristic	Kampo Group (%)	Healthy Group (%)
Total number of patients	10 (100%)	10 (100%)
Sex		
Male	4 (40%)	3 (30%)
Female	6 (60%)	7 (70%)
Diagnosis (Kampo group)		
Head and Neck Cancer	4 (40%)	
Laryngeal cancer	2 (20%)	
Hypopharyngeal cancer	1 (10%)	
Oropharyngeal cancer	1 (10%)	
Parotid gland cancer	1 (10%)	
Breast cancer	6 (60%)	
Adverse effects (Kampo group)	0 (0%)	
Chemotherapy (Kampo group)	6 (60%)	
Radiotherapy (Kampo group)	7 (70%)	

## Data Availability

The original contributions presented in this study are included in the article/Supplementary Materials. Further inquiries can be directed to the corresponding author.

## Supplementary Materials

The following supporting information can be downloaded at: https://www.mdpi.com/article/10.3390/ph18111658/s1, Figure S1: Representative Gating Strategy for NK Cell Subset Analysis by Flow Cytometry. Flow cytometric analysis was performed on peripheral blood mononuclear cells (PBMCs) obtained from a representative cancer patient. (1) From the total cell population, singlet cells were identified by plotting forward scatter height (FSC-H) versus forward scatter area (FSC-A) to exclude doublets and aggregates. (2) Dead cells were eliminated by excluding 7-AAD–positive events. (3) Lymphocytes were then gated from the remaining viable singlet cells based on FSC-A and side scatter area (SSC-A) characteristics. (4) Within the lymphocyte gate, total natural killer (NK) cells were defined as CD3^−^CD56^+^ cells. (5) The NK population was further divided into CD56^dim and CD56^bright subsets, followed by the evaluation of surface marker expression, including CD95, NKp46, NKG2D, and CD161. The plots represent a typical gating strategy from one patient sample before and after Juzentaihoto (JTT) administration; Table S1: Clinical confounding factors among cancer patients treated with Juzentaihoto (JTT). This table summarizes the presence of nine potential confounding factors in ten cancer patients receiving Juzentaihoto (JTT) as part of their treatment. Variables include age > 65 years, prior history of cancer, previous chemotherapy, radiotherapy, history of major or multiple surgeries, comorbidities, low body mass index (BMI < 21), use of immuno-/hormonal drugs, and requirement for enteral feeding via PEG or nasogastric tube. Each item is marked as “TRUE” if the factor is present, accompanied by brief clinical details in parentheses. The final column shows the Confounder Count, indicating the total number of risk factors for each patient. This composite score reflects the degree of baseline clinical complexity that may influence the interpretation of JTT’s therapeutic and immunological effects; Table S2: Changes in the percentage of NK cell subsets and surface markers before and after Juzentaihoto administration analyzed by flow cytometry; Table S3: Changes in mean fluorescence intensity (MFI) of NK cell subsets and surface markers before and after Juzentaihoto administration analyzed by flow cytometry.

## Abbreviations

The following abbreviations are used in this manuscript:

JTT	Juzentaihoto
DNA	Deoxyribonucleic acid
TRAIL	TNF-related apoptosis-inducing ligand
NK	natural killer
BCL-2	B-cell lymphoma 2
FasL	Fas Ligand
PBMCs	Peripheral blood mononuclear cells
OD	optical density
PBS	Phosphate-buffered saline
PEG	Percutaneous Endoscopic Gastrostomy
DISC	Death-Inducing Signaling Complex
TCR	T Cell Receptor
IAP	Inhibitor of Apoptosis Protein
IL-2	Interleukin-2
