# Supplementary material for: Immunomodulatory Effects of Juzentaihoto on Fas-Mediated Apoptosis: Insights from Cancer Patients and In Vitro Models
Source: Pharmaceuticals (Basel). 2025 Nov 1;18(11):1658. doi: 10.3390/ph18111658 (PMC12655088; doi:10.3390/ph18111658)
Supplement: Supplementary file 1 [file pharmaceuticals-18-01658-s001.zip › Supplementary Figure S1.pdf]

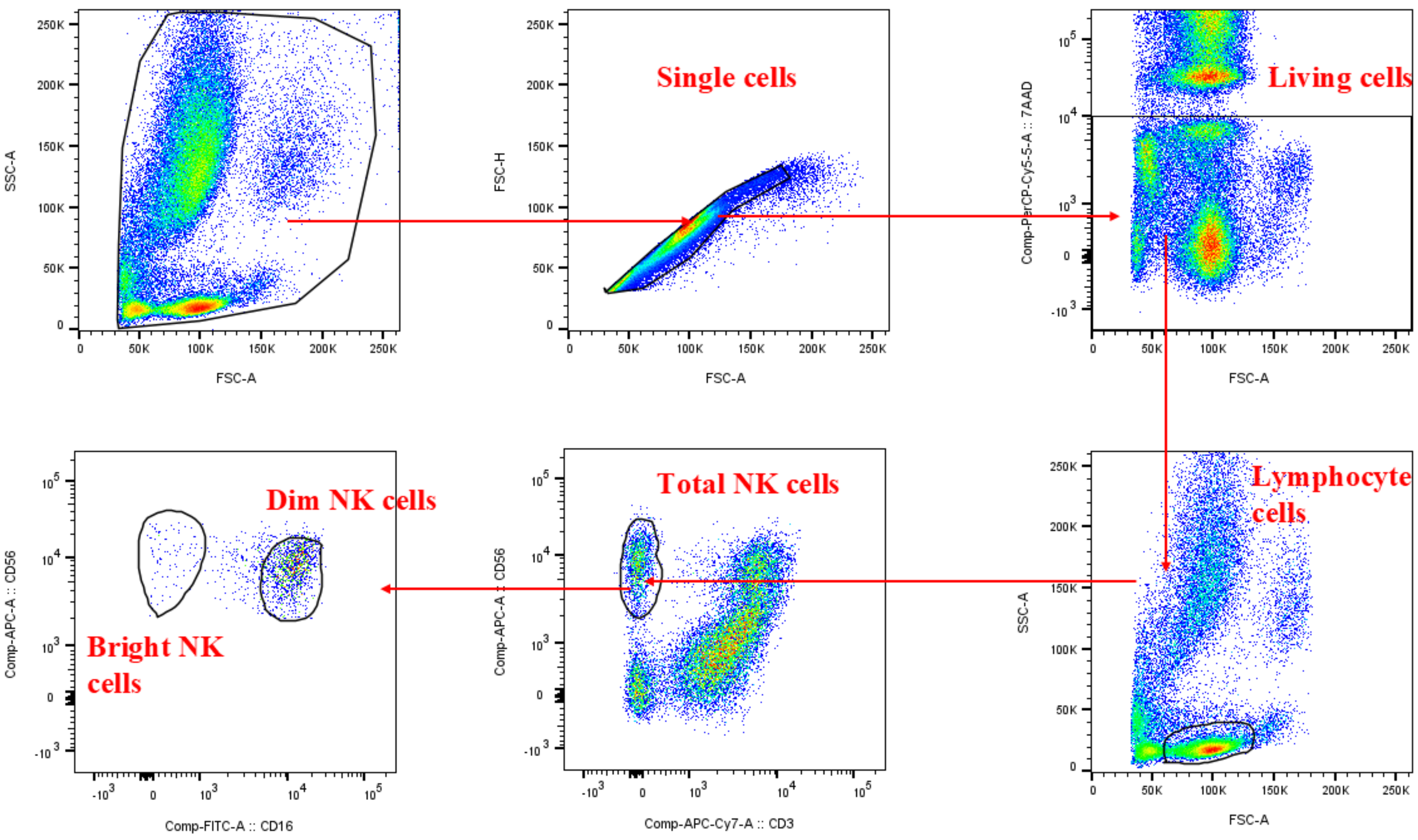

Supplementary Figure S1. Representative Gating Strategy for NK Cell Subset Analysis by Flow Cytometry.

Flow cytometric analysis was performed on peripheral blood mononuclear cells (PBMCs) obtained from a representative cancer patient. (1) From the total cell population, singlet cells were identified by plotting forward scatter height (FSC-H) versus forward scatter area (FSC-A) to exclude doublets and aggregates. (2) Dead cells were eliminated by excluding 7-AAD–positive events. (3) Lymphocytes were then gated from the remaining viable singlet cells based on FSC-A and side scatter area (SSC-A) characteristics. (4) Within the lymphocyte gate, total natural killer (NK) cells were defined as $CD3^{-}CD56^{+}$ cells. (5) The NK population was further divided into CD56^dim and CD56^bright subsets, followed by the evaluation of surface marker expression, including CD95, NKp46, NKG2D, and CD161. The plots represent a typical gating strategy from one patient sample before and after Juzentaihoto (JTT) administration.
