# Supplementary material for: Immunomodulatory Effects of Juzentaihoto on Fas-Mediated Apoptosis: Insights from Cancer Patients and In Vitro Models
Source: Pharmaceuticals (Basel). 2025 Nov 1;18(11):1658. doi: 10.3390/ph18111658 (PMC12655088; doi:10.3390/ph18111658)
Supplement: Supplementary file 1 [file pharmaceuticals-18-01658-s001.zip › Supplementary Table S1.pdf]

Supplementary Table S1. Clinical confounding factors among cancer patients treated with Juzentaihoto (JTT).

This table summarizes the presence of nine potential confounding factors in ten cancer patients receiving Juzentaihoto (JTT) as part of their treatment. Variables include age > 65 years, prior history of cancer, previous chemotherapy, radiotherapy, history of major or multiple surgeries, comorbidities, low body mass index (BMI < 21), use of immuno-/hormonal drugs, and requirement for enteral feeding via PEG or nasogastric tube. Each item is marked as "TRUE" if the factor is present, accompanied by brief clinical details in parentheses. The final column shows the *Confounder Count*, indicating the total number of risk factors for each patient. This composite score reflects the degree of baseline clinical complexity that may influence the interpretation of JTT's therapeutic and immunological effects.

ID	Age > 65	Diagnosis	Prior Cancer	Chemotherapy	Radiotherapy	Major / Multiple Surgeries	Comorbidities	BMI < 21	Immuno-/Hormonal Drugs	PEG / Feeding Support	Confounder Count
1	TRUE (73)	Laryngeal cancer	TRUE (leukoplakia, parotid tumor)	FALSE	TRUE (70 Gy neck)	TRUE (LMS, parotidectomy, etc.)	TRUE (RA, cataract)	FALSE (BMI 22.1)	TRUE (methotrexate, etc.)	FALSE	6
2	TRUE (73)	Right breast cancer	TRUE (thyroid cancer)	FALSE	FALSE	TRUE (mastectomy, thyroidectomy, C-section)	TRUE (post-thyroidectomy)	TRUE (BMI 20.8)	TRUE (levothyroxine, etc.)	FALSE	6
3	FALSE (31)	Left breast cancer	FALSE	TRUE (DOC, FEC, etc.)	TRUE (breast, lymph, spine)	TRUE (multiple surgeries incl. mastectomy, biopsies)	TRUE (depression, disc herniation, etc.)	FALSE (BMI 21.6)	TRUE (TAM, ANA, etc.)	FALSE	5
4	FALSE (50)	Right breast cancer	TRUE (recurrent breast cancer)	TRUE (EC, Letrozole)	TRUE	TRUE (breast surgery, oophorectomy)	TRUE (cardiotoxicity)	FALSE (BMI 23)	TRUE (Letrozole)	FALSE	6
5	TRUE (66)	Right oropharyngeal cancer	FALSE	TRUE (CDDP)	TRUE (70 Gy)	FALSE	TRUE (DM, HT, HL)	FALSE (BMI 24.2)	FALSE	FALSE	4
6	FALSE (46)	Right breast cancer	TRUE (breast cancers)	TRUE (EC → DTX)	TRUE (50 Gy)	TRUE (multiple surgeries for breast cancer)	TRUE (HBOC suspected)	Not Available	TRUE (tamoxifen)	FALSE	6
7	FALSE (54)	Left breast cancer	TRUE (uterine NET, breast cancer)	FALSE	FALSE	TRUE (3 surgeries incl. bowel resection)	TRUE (arrhythmia history)	TRUE (BMI 20.1)	TRUE (anastrozole)	FALSE	5
8	FALSE (61)	Poorly to well differentiated squamous cell carcinoma	TRUE (subglottic cancer)	TRUE (DOC, TS-1)	TRUE (70 Gy)	TRUE (laryngectomy, thyroidectomy)	TRUE (glaucoma, shingles)	TRUE (BMI 19.1)	FALSE	TRUE	7
9	TRUE (76)	Right parotid gland carcinoma	TRUE (bladder cancer)	FALSE	TRUE (66 Gy)	TRUE (parotidectomy, bladder surgery)	TRUE (COPD, etc.)	Not Available	FALSE	FALSE	5
10	TRUE (66)	Hypopharyngeal cancer	TRUE (colon cancer)	TRUE (TS-1)	TRUE (70 Gy + Cetuximab)	TRUE (neck dissection, gastrostomy)	TRUE (HT, renal impairment)	TRUE (BMI 19.9)	TRUE (TS-1)	TRUE	9

Abbreviations: RA, rheumatoid arthritis; DM, diabetes mellitus; HT, hypertension; HL, hyperlipidemia; HBOC, hereditary breast and ovarian cancer syndrome; PEG, percutaneous endoscopic gastrostomy; DOC, docetaxel; DTX, docetaxel; EC, epirubicin/cyclophosphamide; TS-1, tegafur/gimeracil/oteracil; TAM, tamoxifen; ANA, anastrozole; LMS, leiomyosarcoma; NET, neuroendocrine tumor; COPD, chronic obstructive pulmonary disease.
