# Supplementary material for: Immunomodulatory Effects of Juzentaihoto on Fas-Mediated Apoptosis: Insights from Cancer Patients and In Vitro Models
Source: Pharmaceuticals (Basel). 2025 Nov 1;18(11):1658. doi: 10.3390/ph18111658 (PMC12655088; doi:10.3390/ph18111658)
Supplement: Supplementary file 1 [file pharmaceuticals-18-01658-s001.zip › Supplementary Table S2.pdf]

Supplementary Table S2. Changes in the percentage of NK cell subsets and surface markers before and after Juzentaihoto administration analyzed by flow cytometry.

Marker	Baseline Percentage Mean±SD	After Percentage Mean±SD	Δ Mean Percentage	95% CI (Percentage)	p-value (Percentage)
**CD16+CD56^ dim NK cell**	11.15±6.04	16.16±7.74	5.0100	[-2.276, 12.296]	0.154237
**CD16-CD56^ bright NK cell**	4.86±4.90	5.06±4.63	0.2000	[-4.781, 5.181]	0.92962
**NKp46 +NK cell**	33.53±12.94	37.67±13.02	4.1403	[-3.871, 12.151]	0.272386
**NKG2D+ NK cell**	66.37±7.66	61.11±9.67	-5.2631	[-10.717, 0.191]	0.056905
**CD161+ NK cell**	22.81±10.47	18.70±10.93	-4.1053	[-8.667, 0.457]	0.072273
**CD11a + NK cell**	99.46±0.92	99.36±1.20	-0.1036	[-1.053, 0.846]	0.810649
**CD95+ NK cell**	64.91±15.43	55.58±19.64	-9.3337	[-16.307, -2.360]	**0.014298***

Note: Data are expressed as mean ± standard deviation (SD) for ten patients at baseline (day 0) and after 14 days of Juzentaihoto administration.
