# Supplementary material for: Immunomodulatory Effects of Juzentaihoto on Fas-Mediated Apoptosis: Insights from Cancer Patients and In Vitro Models
Source: Pharmaceuticals (Basel). 2025 Nov 1;18(11):1658. doi: 10.3390/ph18111658 (PMC12655088; doi:10.3390/ph18111658)
Supplement: Supplementary file 1 [file pharmaceuticals-18-01658-s001.zip › Supplementary Table S3.pdf]

Supplementary Table S3. Changes in mean fluorescence intensity (MFI) of NK cell subsets and surface markers before and after Juzentaihoto administration analyzed by flow cytometry.

Marker	MFI Baseline Mean±SD	MFI After Mean±SD	MFI Δ Mean	MFI 95% CI	MFI p-value
**CD16+CD56^ dim NK cell**	17341.50±6536.25	15395.10±7259.71	-1946.4	[-8452.86, 4560.06]	0.515
**CD16-CD56^ bright NK cell**	275.89±224.35	493.24±356.39	217.35	[-70.06, 504.76]	0.121
**NKp46 +NK cell**	2450.24±1007.95	2465.03±701.74	14.78938	[-461.548, 491.126]	0.945542
**NKG2D+ NK cell**	4051.44±857.63	3409.50±652.53	-641.935	[-1226.894, -56.976]	**0.034849***
**CD161+ NK cell**	1336.22±606.91	1255.01±578.55	-81.2111	[-370.326, 207.904]	0.540965
**CD11a + NK cell**	140266.28±20855.71	129420.71±20544.92	-10845.6	[-33997.815, 12306.681]	0.316892
**CD95+ NK cell**	3709.00±1850.58	2981.63±1194.48	-727.374	[-1313.318, -141.430]	**0.020438***

Note: Data are expressed as mean ± standard deviation (SD) for ten patients at baseline (day 0) and after 14 days of Juzentaihoto administration.
