# Supplementary figures and images for: Immunomodulatory Effects of Juzentaihoto on Fas-Mediated Apoptosis: Insights from Cancer Patients and In Vitro Models
Source: Pharmaceuticals (Basel). 2025 Nov 1;18(11):1658. doi: 10.3390/ph18111658 (PMC12655088; doi:10.3390/ph18111658)

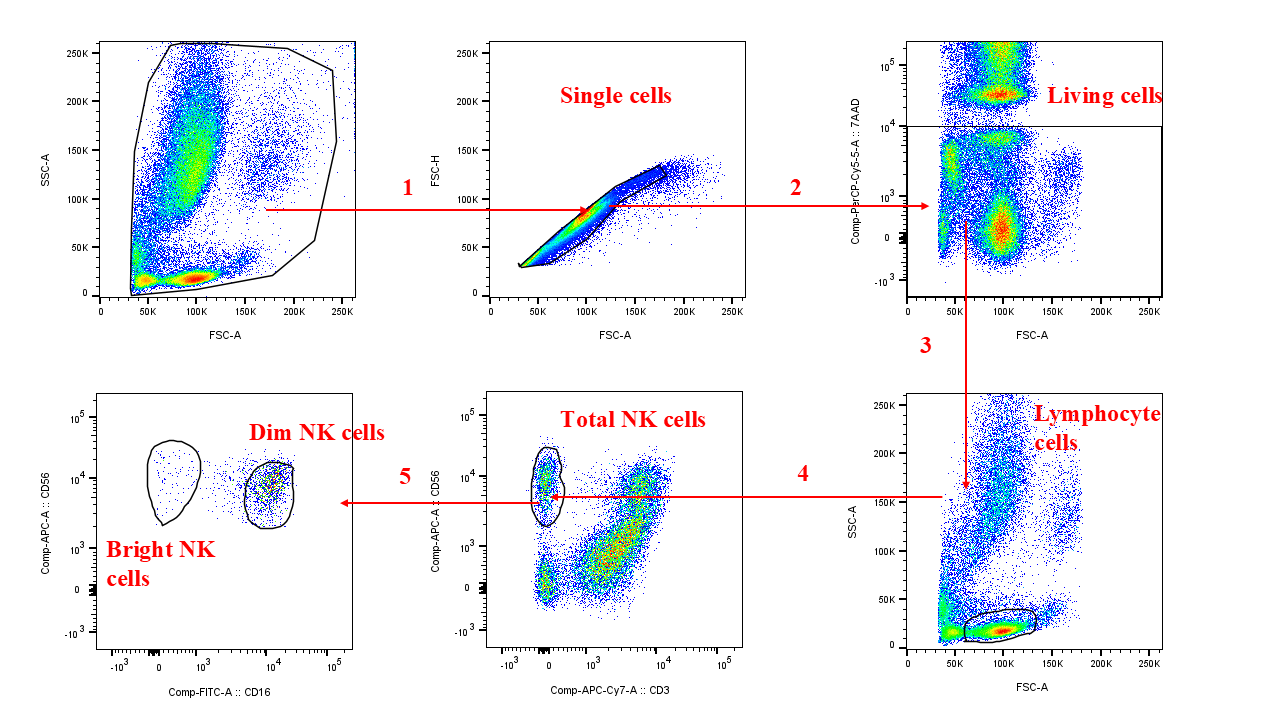

Supplement: Supplementary file 1 [file pharmaceuticals-18-01658-s001.zip › Supplementary Figure S1.tif]
